# A Rare Presentation of Systemic Lupus Erythematosus in a Patient With Fever of Unknown Origin

**DOI:** 10.7759/cureus.59286

**Published:** 2024-04-29

**Authors:** Eric Kawana, Rodd Rahmani, Scott Turnbull, Adam Khattak, Kenny H Do, Aditi Singh

**Affiliations:** 1 Department of Internal Medicine, Kirk Kerkorian School of Medicine at University of Nevada Las Vegas (UNLV), Las Vegas, USA

**Keywords:** sytemic lupus erythematosus, adult-onset still’s disease, autoinflammatory disease, macrophage activation syndrome (mas), differential for fever of unknown origin

## Abstract

This case presents a 23-year-old male with a rare presentation of lupus as fever of unknown origin (FUO). The patient's clinical symptoms, examination findings, and laboratory results painted a complex picture that necessitated considering macrophage activation syndrome and adult-onset Still's disease but ultimately led to the diagnosis of systemic lupus erythematosus. The case emphasizes the importance of including lupus in the differential diagnosis of FUO given the associated risks and higher mortality rates in this demographic, especially in males. Understanding lupus prevalence and classification criteria aids in diagnosis, highlighting the importance of a systematic approach for FUO and emphasizing timely intervention for improved patient outcomes.

## Introduction

The description of unexplained fevers dates back to ancient medical writings which show these fevers to have occurred since as early as human existence [1-2]. However, it was not until the mid-20th century that the term "fever of unknown origin" (FUO) started to be used. Coined by Dr. Petersdorf and Dr. Beesom in 1961, they described it as a temperature of 101 degrees Fahrenheit or higher, lasting for at least three weeks without a confirmed diagnosis, even after intensive investigation [3].

Since then, the diagnostic criteria for FUO have evolved over the years to accommodate current medical advancements. An inpatient workup lasting one week with no clear diagnosis was deemed necessary to diagnose FUO. However, with the current technology allowing more advanced outpatient evaluations, these criteria have fallen out of favor. In addition, the definition has broadened to also include temperatures higher than 38.3°C on multiple occasions [4].

It is known that there are over 200 known possible causes of FUO [5]. Since fevers tend to be nonspecific yet highly prevalent, FUO can be a diagnostic dilemma for clinicians. To make things even more difficult, algorithms are scarce, and the few algorithms available to diagnose FUO can be vague; furthermore, there may only be a handful of clues that indicate or rule out specific diagnoses.

Lupus, a complex autoimmune disease, presents a multifaceted clinical picture often characterized by its diverse array of symptoms. Among these symptoms, FUO stands out as a significant manifestation in a subset of lupus patients [6]. In this report, we describe a patient with a rare presentation of a lupus flare as a presentation of FUO.

## Case presentation

A 23-year-old male with no significant medical history presented to the emergency department (ED) with one week of fever, cough, and worsening shortness of breath. He reported a maximum self-recorded temperature of 101°F and was treated with ibuprofen which did not improve his symptoms. Outpatient evaluation included two negative at-home COVID tests and negative influenza. The patient reported night sweats but denied any nausea, vomiting, diarrhea, constipation, or sore throat. He denied any known sick contacts. He further denied any significant family history and denied using cigarettes or consuming alcohol.

Upon assessment, he had a temperature of 39.2 degrees Celsius, a heart rate of 113, a respiration rate of 22, and a BMI of 38.08 kg/m^2^. An oral ulcer was observed on the left buccal mucosa. No cervical or axillary lymphadenopathy was noted. His breath sounds were clear on auscultation, and no audible wheezing, rales, or rhonchi were heard. Physical examination was otherwise unremarkable. 

Examination

Initial laboratory findings (Table 1) revealed WBC of 2.99/μL, platelets at 90/μL, and elevated liver enzymes (ALT: 209 U/L, AST: 175 U/L, alkaline phosphatase: 191 U/L). Lactic acid levels were normal (1.01). Initial ESR and CRP were 21 mm/hr and 98.96 mg/dl, respectively. He was also noted to have other significantly elevated inflammatory markers, including ferritin (1,404.8 ng/ml) and D-dimer (3.47 mg/l). ESR measured 51 mm/hr four days later, while CRP measured 102.68 mg/dl two days afterward. Urinalysis showed 5-10 RBCs with 2+ blood and 1+ protein. Further investigation through lab work yielded inconclusive results (normal IgG, IgA, IgM, negative respiratory BioFire, chlamydia, gonorrhea, lipase, streptococcal and Legionella urinary antigen, EBV, acute hepatitis panel, QuantiFERON TB gold, C3 and C4 complement levels, syphilis antibody, malaria smear, AFP tumor marker, blood cultures, urine cultures) and negative for infectious causes (HIV, influenza, respiratory pathogens). 

**Table 1:** Laboratory Values at the Time of Admission ALT: Alanine transaminase; AST: aspartate aminotransferase

	Result	Reference Range
SERUM		
Hepatic		
ALT	209 U/L	7-55 U/L
AST	175 U/L	8-48 U/L
Alkaline phosphatase	191 U/L	44-147 U/L
Other		
Lactic Acid	1.01mmol/L	<2 mmol/L
Ferritin	1,404.8 ng/ml	12-300 ng/ml
HEMATOLOGIC		
Leukocyte Count (WBC)	2.99 × 10^9^ /L	4.5-11 × 10^9^ /L
Platelet Count	90/μL	150-400/μL
Other		
Erythrocyte Sedimentation Rate (ESR)	21 mm/hr	<15 mm/hr
C-Reactive Protein (CRP)	98.96 mg/dL	<0.3 mg/dL
D-Dimer	3.47 mg/L	<0.5 mg/L
URINALYSIS		
RBC	5-10/Hpf	0-4/Hpf
Blood	2+	Negative
Protein	1+	Negative

He had multiple imaging studies including CT scans of the chest and abdomen which demonstrated no remarkable source of infection or malignancy, though hepatosplenomegaly was noted. The patient presented with a cholestatic pattern of liver injury, with hepatomegaly and steatosis observed on ultrasound. Abdominal Doppler ultrasound ruled out pylephlebitis, and the liver abnormalities were attributed to multifactorial liver injury related to autoimmune etiology and obesity. Tumor markers and testicular ultrasound were normal. A CT angiogram was negative, and the patient's shortness of breath was regarded as unlikely due to pneumonia or pulmonary embolism.

Further serological studies revealed a positive antinuclear antibody (ANA) (speckled pattern) with a titer of 1:320. Other antibodies were largely unremarkable (DS-DNA antibody, anti-RNP, anti-Smith, Scleroderma antibody, SSA/SSB antibody, Jo-1 antibody, RF, pANCA, cANCA, anti-LKM, anti-Smooth muscle, anti-mitochondrial) leading to a likely diagnosis of systemic lupus erythematosus (SLE) flare. During his nine-day stay at the hospital, the inflammatory markers and liver function tests spontaneously normalized, along with the resolution of cyclical fevers on the fifth day. Additionally, both leukopenia and thrombocytopenia resolved within a span of two days. On his last day, the patient was considered to be stable and, prior to discharge, he was started on a daily regimen of 400 mg hydroxychloroquine, along with a gradually decreasing prednisone taper. Subsequent outpatient follow-ups with a rheumatologist were planned to monitor his progress.

Differential diagnosis

The patient presented with a fever of undetermined cause. The fever was accompanied by abnormal blood markers including elevated ferritin levels, elevated liver enzymes, elevated CRP, elevated LDH, elevated D-dimer, and initial pancytopenia. In conjunction with hepatosplenomegaly, this overall picture was suggestive of macrophage activation syndrome (MAS). The notable elevation in ANA titer further suggested probable SLE with features of MAS.

Adult-onset Still's disease (AOSD) was also considered. The one deterring factor was the absence of initial leukocytosis, as pancytopenia would present in more severe MAS cases associated with AOSD. Improving thrombocytopenia and leukopenia along with unremarkable imaging decreased the likelihood of a hematologic malignancy. Although the respiratory viral panel and CMV/EBV PCR were negative, an explanation for this patient’s clinical presentation was a resolving viral infection that triggered a lupus flare with features of MAS.

## Discussion

SLE is a chronic autoimmune disease that affects multiple body parts. In patients with the disease, the immune system mistakenly attacks healthy tissues [7]. The exact cause of lupus is not fully understood, but it is believed to involve a combination of genetic and environmental factors.

In 1991, Durack and Street proposed a revised classification, categorizing FUO cases into four subclasses: classic, nosocomial, neutropenic, and HIV-related (Table 2) [8]. The four subgroups of the differential diagnosis of FUO were further specified to include infectious, neoplastic, non-infectious inflammatory diseases, and miscellaneous [4]. Some common infectious etiologies include miliary tuberculosis (TB), brucellosis, and Coxiella burnetii infection (Q fever) [5]. The most common neoplastic causes of FUO are lymphoma and renal cell carcinoma. The most common non-infectious inflammatory causes of FUO tend to be autoimmune conditions such as AOSD and giant cell arteritis. In this report, we describe a patient with a rare presentation of a lupus flare as a presentation of FUO. This case of a 23-year-old male highlights an initially undetermined cause of fever that ultimately led to a diagnosis of SLE.

**Table 2:** Categorization of Fever of Unknown Origin (FUO)

Category of FUO	Definition	Examples
Classic	Fever persisting for over three weeks without a determined cause following a three-day hospital evaluation or at least three outpatient visits.	Lymphoma, Renal Cell Carcinoma, Miliary Tuberculosis, Adult Still’s Disease, Giant Cell Arteritis
Nosocomial	Fever in hospitalized patients with an uncertain diagnosis after three days of thorough evaluation, and no existing or incubating infection at admission.	Clostridium difficile colitis, Pulmonary Embolism, Septic Thrombophlebitis
Neutropenic	Fever in neutropenic and immunodeficient patients, with an uncertain diagnosis after three days of thorough evaluation.	Aspergillosis, Candidemia, Opportunistic Bacterial Infections
HIV-Related	Fever lasting over three days in inpatient HIV patients, with an uncertain diagnosis after thorough evaluation.	Pneumocystis Carinii Pneumonia, Kaposi’s Sarcoma, Cytomegalovirus

A recent assessment of lupus incidence by the CDC in the United States indicates a rate of 5.1 cases per 100,000 person-years, considerably higher in women than in men (8.7 and 1.2, respectively) [9]. Among women, the highest incidence is observed in Black individuals (15.9), followed by Asian/Pacific Islander (7.6), Hispanic (6.8), and White (5.7) women. The incidence in men is highest among Black individuals (2.4), followed by Hispanic (0.9), White (0.8), and Asian/Pacific Islander (0.4) men. 

The classification for SLE according to ACR criteria requires positive ANA as a criterion [10]. Additional criteria include seven clinical categories (constitutional, hematologic, neuropsychiatric, mucocutaneous, serosal, musculoskeletal, renal) and three immunologic categories (antiphospholipid antibodies, complement proteins, SLE-specific antibodies), each weighted from 2 to 10. A total score of at least 10 is required to classify SLE. The presence of leukopenia (3), thrombocytopenia (4), fever (2), and oral ulcers (2) amount to a score of 11, thus affirming the diagnosis of lupus in this patient. 

Studies show that males with lupus are at higher risk for developing end-stage renal disease, cardiovascular disease, and thromboembolic events, all giving rise to higher mortality rates in patients who fit this demographic [11-12]. For this reason, it is imperative to include lupus in the list of differential diagnoses for patients exhibiting FUO and symptoms resembling SLE. After diagnosis, prompt treatment and detection of organ involvement are crucial in improving prognosis. 

## Conclusions

In conclusion, this case emphasizes the complexity of FUO, revealing a likely lupus flare as the underlying cause. Understanding the prevalence and classification criteria for lupus contributes to the diagnostic process. The case report emphasizes the need to consider autoimmune disorders, such as lupus, as well as a systematic approach in the context of FUO. An awareness of lupus-related complications, especially in males, underscores the importance of a punctual diagnosis and subsequent intervention to enhance patient outcomes.
